# Heat Stress Challenges in Marathon vs. Ultra-Endurance Running

**DOI:** 10.3389/fspor.2019.00059

**Published:** 2019-11-13

**Authors:** Nicolas Bouscaren, Guillaume Y. Millet, Sebastien Racinais

**Affiliations:** ^1^Inserm CIC1410, CHU Réunion, La Réunion, Saint Pierre, France; ^2^Univ Lyon, UJM-Saint-Étienne, Inter-University Laboratory of Human Movement Biology, EA 7424, Saint-Étienne, France; ^3^Aspetar Orthopedic and Sports Medicine Hospital, Doha, Qatar

**Keywords:** thermoregulation, ultra-endurance, temperature, hyperthermia, exercise in the heat

## Abstract

Several studies have investigated the effect of hot and humid ambient conditions on running exercise up to the marathon. However, studies on exercise longer than marathon are sparse. Events exceeding 6 h can be defined as ultra-endurance and have variable characteristics (e.g., distance, elevation profile, technical difficulty, altitude, night running) making hazardous the transposition of the current knowledge obtained in marathon to ultra-endurance running. Thus, the aim of this manuscript was to discuss the potential differences between marathon and ultra-endurance running in terms of heat stress challenges. The high running intensity (especially for the fastest runners), the urban context with high albedo effect materials, and the hot self-generated microclimate in mass-participation events (especially for the average to slow runners) are specific risk factors associated with marathon running in hot environments. Uphill running/walking (sometimes with poles), exotic destination with long-haul travel, desert environment and the necessity to sustain thermoregulatory and sweating responses for several days are risk factors more specific to ultra-endurance running. These differences call for specific research on the effect of hot ambient conditions in ultra-endurance disciplines to create appropriate recommendations.

## Introduction

The state of Qatar will host the 17th International Association of Athletics Federations (IAAF) World Championships from the 27th of September to the 6th of October 2019. The track and field events will be held in an air-conditioned stadium, whilst the road events (i.e., marathon and race-walk) will likely be held in hot and humid ambient conditions. World Championships in athletic and other summer sports are often performed in hot conditions. Thus, several studies have considered the effect of such conditions on running event up to the marathon (Cheuvront and Haymes, 2001; Kenefick et al., 2007; Bergeron, 2014). Hot ambient conditions are known to impair performance and increase the risk of heat illness in “classic” endurance events (Maughan, 2010; Racinais et al., 2015). The decrease in performance is linked to the event duration with a larger decrease during marathon than track and field events (Guy et al., 2015). This suggests that the longer the event, the larger the effect of ambient conditions. One could extrapolate that ultra-endurance running would therefore be more affected than marathon running. Ultra-endurance can be defined as an event with a winning time exceeding 6 h (Zaryski and Smith, 2005; da Fonseca-Engelhardt et al., 2013). There has been a marked increase in the number of participants in ultra-endurance running in the past two decades (Hoffman et al., 2010; da Fonseca-Engelhardt et al., 2013; Cejka et al., 2014; Scheer, 2019), and some events such as the Marathon des Sables, the Western States Endurance Run, the Bad Water Race, or the Grand Raid Reunion are held in extreme hot and humid ambient conditions (temperature >30°C and/or humidity >70%). However, even if ultra-endurance races are longer, exercise intensity may be a more potent determinant of body temperature than exercise duration (Racinais et al., 2019). Thus, ultra-endurance running may have lower thermoregulatory requirements than marathon due to their lower average intensity, albeit those requirements need to be sustained for a longer period of time. In addition, natural environments in ultra-trail running (i.e., ultra-endurance competition running in natural environment with lower than 20% of cemented or asphalted road) are also more diverse between and within events as compared with most city marathons. In summary, the characteristics of ultra-endurance (e.g., distance, elevation profile, technical difficulty, altitude, day, vs. night running) make it difficult to extrapolate the current knowledge obtained in distances shorter than, or equal to, marathon to ultra-endurance (Bergeron, 2014). Therefore, the aim of this manuscript is to explain the need for specific research on the thermoregulatory requirements in ultra-endurance events. This is not a comprehensive review of the existing knowledge on thermoregulatory responses during marathon or other activities, but rather a reflection on the potential specificities of ultra-endurance and their associated research requirements.

## Metabolic Heat Production and Core Temperature

Muscle efficiency in using the energy released by hydrolyzing adenosine triphosphate is about 20–25%, i.e., about 75–80% does not contribute to external work and is internally released as heat (González-Alonso et al., 2000; González-Alonso, 2007; Lim et al., 2008). This metabolic heat production needs to be dissipated to the environment in order to limit the increase in core temperature. Several factors affect the balance between heat production (e.g., exercise intensity) and dissipation (e.g., environmental heat and humidity, skin temperatures, and wetness). During ultra-trail, hill running and the use of trekking poles increase muscle recruitment and impair running economy, consequently resulting in increase heat production for a given running speed (Christensen and Ruhling, 1980; Mora-Rodriguez et al., 2011). Furthermore, the low running pace and the long sections of walking during ultra-endurance running limit self-generated wind velocity and convective cooling when compared with marathon running (Mora-Rodriguez et al., 2011). However, the main factor for heat production is exercise intensity (Racinais et al., 2019) and longer events may be less prone to hyperthermia in a given environment, as they are performed at a lower intensity. For example, a study on 31 heat-acclimated male soldiers participating in a half marathon in tropical environment revealed that 68% of the finishers completed the race with a gastrointestinal temperature > 40°C (Lee et al., 2010), while in a 142 km trail run performed over 6 days in tropical environment, the maximal gastrointestinal temperature was only 38.3–38.7°C (Hue et al., 2014). These findings warrant specific field research during ultra-endurance events in hot and humid ambient conditions to characterize the thermal responses to various race situations. In the meantime, we hypothesize that the moderate intensity during ultra-endurance events for most recreational athletes limits the risk of exertional heat stroke.

## Performance

Athletic performance can be influenced both positively (sprint) and negatively (middle—long distances) by hot compared with temperate climate. Indeed, whereas an increase in muscle temperature benefits performance during explosive efforts, an increase in core temperature may impair performance during prolonged exercise in hot and/or humid environments by challenging the circulatory system. Briefly, the narrowing of the core-to-skin temperature gradient lead to a redistribution of the blood flow toward the skin for heat dissipation, and the subsequent decrease in ventricular filling pressure reducing stroke volume is exacerbated by an intrinsic increase in heart rate. Moreover, the necessity to sustain an elevated sweat rate for heat dissipation may lead to dehydration if fluids are not replaced. Thus, during the IAAF world championships held with an ambient temperature above 25°C, an ~2% decrease in performance was reported for distances longer than 5,000 m as compared to the other editions, this difference being as much as 3% in marathon for male athletes (Guy et al., 2015). The decrement in marathon performance exists for both men and women of various performance levels although the slowest runners are more affected by the heat than their faster counterparts (Ely et al., 2007, 2008; Vihma, 2010). In ultra-endurance, a decrease in performance of ~8% has been reported in participants competing in the Western States Endurance Run (161 km and 6,000 m of cumulative climb) during a hot edition (2006, temperature ranging from 7.2 to 38.0°C), compared with a cooler edition (2007, temperature ranging from 2.2 to 30.6°C) (Parise and Hoffman, 2011). Conversely to marathon, the performance of the slowest ultra-runners was less impacted by hot conditions compared with the fastest ones (Parise and Hoffman, 2011). This was partially explained by the fact that slower runners ran more overnight, at a time of lower heat stress, than their faster counterparts in this ultra-endurance races starting early in the morning. Slow ultra-runners may also have less technical abilities, limiting their ability to run fast, and thus produce heat on an uneven terrain. An explanation for the greater performance changes in the heat for slow marathon runners could be the hot microclimate generated by large groups of runners close to each other during mass participation events (De Freitas et al., 1985). In this condition, radiant and convective heat losses are limited within the group, resulting in excessive heat load during the race, directly affecting performance. Although ultra-endurance is expanding rapidly, it is not as popular compared with the largest marathon in terms of number of participants (e.g., 49,155 runners in Paris marathon in 2019 vs. 2,300 runners in the Ultra-Trail du Mont-Blanc). Thus, in ultra-trail, except at the start, the crowd is traditionally less dense and spread over a longer distance, with most of the race performed in a single file on narrow pathways. This likely limits the hot microclimate generated by large groups as observed in marathons.

## Environment and Characteristics of Races

**Figure 1:**
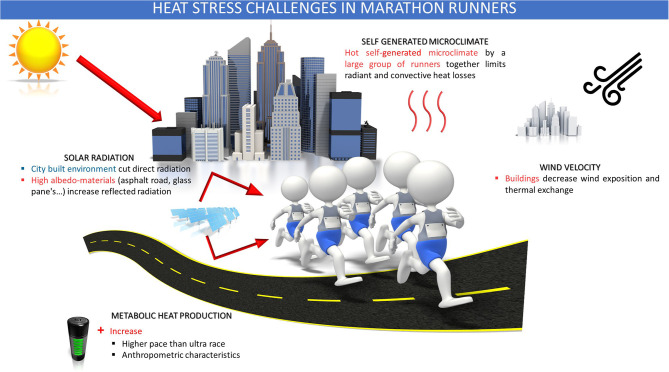
Heat stress challenges in marathon runners (adapted with permission from http://www.ephysiol.com/).

The most famous and popular marathons are held in western city environment (Boston, Chicago, London, New York, Paris). The shade from the built environment (building and trees) produces low sky view factors over roads and protects the runners from the direct radiant and diffuse radiation heat (Kenny et al., 2008; Lai et al., 2017). Inversely, the usage of high albedo-materials (asphalt road, glass panes…) could impact the urban heat island in street canyons and increase overall thermal stress (Erell et al., 2014; Middel et al., 2014). The lower wind velocity in cities due to the surrounding buildings and constructions also impairs heat dissipation (Figure 1).

**Figure 2:**
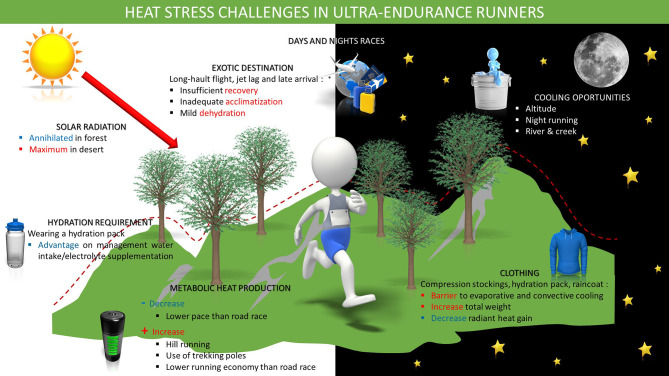
Heat stress challenges in ultra-endurance runners (adapted with permission from http://www.ephysiol.com/).

Conversely, ultra-endurance races (particularly ultra-trail running) traditionally take place in natural environments (mountain, desert, forest…) with large positive and negative elevation. The regulation of trail running states that road sections should not exceed 20% of the total course (ITRA, 2019). The diversity of natural environments in ultra-trail running makes the study to heat and radiation exposure more difficult. For example, sky view factor, and direct radiation can be completely absent when running in a forest vs. maximum in deserts such as the multistage ultramarathon event called the “marathon des sables.” Weather conditions vary as much as the course topography and can change considerably from start to finish for a given race (night and day running, altitude, temperature, precipitations…). As mentioned above, temperatures ranged from 7.2 to 38.0°C in the 2006 Western States Endurance Run (Parise and Hoffman, 2011). The Grand Raid de la Reunion is a race starting and finishing at sea level with temperatures and relative humidity often exceeding 30°C and 80%, respectively; while other sections are ran at an altitude above 2,000 m where temperatures can be negative (Association GRR, 2019; Lai-Cheung-Kit et al., 2019). These variations may be viewed as a physiological constraint, but the low temperatures at altitude and during the nights may also minimize the level of heat stress during ultra-endurance races as compared with the marathon (Bongers et al., 2015; Tyler et al., 2015; Figure 2).

In addition, ultra-endurance events are commonly held in exotic destinations, with ultra-endurance runners being more motivated by nature and life experience than the competition itself (Doppelmayr and Molkenthin, 2004; Waśkiewicz et al., 2018). Such destinations may necessitate to long-haul flight and jet lag, with amateur participants arriving only a few days before the race. This often prevents enough recovery and acclimatization to the local conditions. Ideally, the heat acclimatization period should last 2 weeks in order to maximize adaptations (i.e., decreases in heart rate, skin and rectal temperature, increases in sweat rate, and work capacity) and limit the impact of exercising in a warm environment (Périard et al., 2015; Racinais et al., 2015). Furthermore, long-haul flights can induce mild dehydration from insufficient fluid intake, consumption of diuretic beverages, and low ambient humidity (Hamada et al., 2002) despite the recommendation to ensure euhydration before exercising in hot environments (Racinais et al., 2015).

## Clothing

Clothing substantially differs between the two disciplines. Marathon runners traditionally wear minimalist and light textiles: shorts, t-shirts, or tank tops. Trail runners traditionally wear a more complex outfit including shorts with underneath compression shorts (to limit irritation due to long distance), compression stockings, a hydration pack, and a headscarf or a cap. It is also often necessary/mandatory in ultra-endurance races to carry warm clothing, gloves and a raincoat to anticipate changes in environmental conditions. This increases total weight and thus thermal stress in the warmest sections of the race. Clothing creates a microenvironment between the skin and clothing (Bishop et al., 2000). Clothing can act as a protective function by reducing radiant heat gain and thermal stress (Gavin, 2003). Exercise in the shade as opposed to in the sun reduces radiant heat gain as much as 100 W and decreases the need for evaporative heat loss (Nielsen et al., 1988; Gavin, 2003). In this context semi-nude (short, socks, and shoes) exercise during marathon is a disadvantage compared with ultra-endurance considering radiant heat load. On the other hand, clothing and gear represents a layer of insulation and act to inhibit evaporative and convective cooling, and could significantly minimize thermoregulatory capacities of ultra-endurance runners (Davis and Bishop, 2013; Davis et al., 2017). Indeed, the evaporation of sweat from the skin surface is the main modifiable avenue of heat lost (Périard and Racinais, 2015). Then, the insulative effect of clothing represents a significant restriction on heat dissipation compared to the protective function against radiant heat gain. Finally, color of clothing can affect radiative heat gain, where white clothing reduces it compared with black clothing (Shkolnik et al., 1980; Nielsen, 1990), but no clothing color habits are specific to a discipline to our knowledge.

## Hydration Requirement and Dehydration

Sufficient hydration prior to, during, and after the race is crucial for athletic performance and safety during training and competition in the heat (Bergeron et al., 2012; Racinais et al., 2015). Athletes should avoid body water deficits exceeding 2% of their body mass during exercise in order to prevent an impairment of thermoregulatory function, an elevation of cardiovascular strain and an impairment of aerobic exercise performance in many conditions (e.g., warmer, longer, more intense) (Valentino et al., 2015; Kenefick, 2018). However, in a 161-km ultra-marathon, it has been established that runners tend to lose between 2 and 4% without core temperature elevation or consequences on performance (Lebus et al., 2010; Hoffman and Stuempfle, 2014; Valentino et al., 2015). A review of fluid balance data from marathon running literature provides average dehydration values of 3.2% in cool weather to 4% in warm weather (Cheuvront and Haymes, 2001). Actually the level of sustainable dehydration and hydration guidelines during endurance are a much-debated topics (Wall et al., 2015; Kenefick, 2018). Any ways, ultra-endurance athletes adopt hydration and sodium supplementation strategies, just as endurance athletes do for fluid replacement management. Nevertheless, several differences can exist between ultra-endurance and marathon and the long duration of the ultra-endurance events may create sport-specific hydration issues:

Firstly, while marathon runners have only opportunity to rehydrate at drink stations, ultra-endurance athletes have regular access to fluids by wearing a hydration pack or belt. In ultra-endurance, carrying your gear may help with regular hydration but also impair evaporation and convective cooling.

Secondly, gastro-intestinal (GI) distress may impair athlete ability to feed and hydrate adequately in both disciplines. The prevalence of GI symptoms ranges from 4 to 52% among marathon runners (Rehrer et al., 1989; Halvorsen et al., 1990; Costa et al., 2017; Pugh et al., 2018), but reaches 35–96% among ultra-runners (Stuempfle and Hoffman, 2015; Wardenaar et al., 2015; Stuempfle et al., 2016). These GIs symptoms, and particularly nausea and vomiting if the fluid losses are not compensated, could lead to and worsen progressive dehydration and impair thermoregulation. The reduction of blood flow and then whole-body sweat rate decrease heat loss, thus accounting for the increase in core temperature (American College of Sports Medicine et al., 2007).

Finally, in conjunction with hydration level and sodium supplementation; the occurrence and incidence of Exercise Associated Hyponatremia (EAH) varies with exercise type and duration, as well as the level of heat stress during the event (Hew-Butler et al., 2015). The incidence of EAH has been largely investigated in marathoners, ranging from 0 to 12–13% of races finishers and even under cool conditions (Reid et al., 2004; Almond et al., 2005; Mettler et al., 2008; Kipps et al., 2011). In ultra-endurance incidence is highly variable between studies and ranges between 5 and 51% of runners (Lebus et al., 2010; Hoffman et al., 2012, 2013; Cairns and Hew-Butler, 2015). These important variations in EAH prevalence between studies could be due to several factors such as exercise intensity or duration, hydration strategies (overhydration), salt supplementation, and environmental temperature and hygrometry (Knechtle and Nikolaidis, 2018).

## Perspectives

The purpose of this manuscript was to present some potential differences between marathon and ultra-endurance running in terms of heat stress challenges. The high running intensity (especially for the fastest runners), the urban context with high albedo effect materials, and the hot self-generated microclimate in mass-participation events (especially for the average to slow runners) are the main specific risk factors for high heat load associated with marathon running in hot environments. Uphill running/walking (sometimes with poles), lower self-generated wind velocity, exotic destination with long-haul travel, desert environment and the necessity to sustain a thermoregulatory and sweating responses for days represent some of the risk factors more specific to ultra-endurance. Based on these differences, it appears difficult to extrapolate our knowledge from traditional marathon to ultra-endurance events, and specific research appeared to be warranted. Future research should notably determine the thermoregulatory responses to ultra-endurance events lasting for a day or more and the effect of changing of environment within the same events. It is also important to determine the hydration requirements in such conditions along the associated risk for dehydration, gastrointestinal problems, and hyponatremia. Lastly, the impact of clothing and gear (e.g., back-pack, lights, and other mandatory equipment for ultra-endurance event) on thermoregulation should also be considered.

## Author Contributions

All authors listed have made a substantial, direct and intellectual contribution to the work, and approved it for publication.

### Conflict of Interest

The authors declare that the research was conducted in the absence of any commercial or financial relationships that could be construed as a potential conflict of interest.
